# Clonal Complex 12 Serotype Ib Streptococcus agalactiae Strain Causing Complicated Sepsis in Neonates: Clinical Features and Genetic Characteristics

**DOI:** 10.1128/spectrum.03778-22

**Published:** 2022-12-08

**Authors:** Jen-Fu Hsu, Yu-Ning Chen, Shih-Ming Chu, Wei-Ju Lee, Hsuan-Rong Huang, Ming-Chou Chiang, Peng-Hong Yang, Ming-Horng Tsai, Jang-Jih Lu

**Affiliations:** a Division of Pediatric Neonatology, Department of Pediatrics, Linkou Chang Gung Memorial Hospital, Taoyuan, Taiwan; b School of Medicine, College of Medicine, Chang Gung University, Taoyuan, Taiwan; c Division of Neonatology and Pediatric Hematology and Oncology, Department of Pediatrics, Yunlin Linkou Chang Gung Memorial Hospital, Yunlin, Taiwan; d Department of Laboratory Medicine, Linkou Chang Gung Memorial Hospital, Taoyuan, Taiwan; e Department of Medical Biotechnology and Laboratory Science, Chang Gung University, Taoyuan, Taiwan; University of Brescia

**Keywords:** group B *Streptococcus*, serotype Ib CC12 GBS, multilocus sequence typing, antimicrobial resistance, severe sepsis

## Abstract

Streptococcus agalactiae (group B Streptococcus [GBS]) is well known to cause serious diseases in infants. A serotype Ib GBS strain has recently emerged and become prevalent in Southeast Asia. We aimed to investigate the clinical and genetic characteristics of this strain. All neonates with invasive GBS diseases from a tertiary-level medical center in Taiwan between 2003 and 2020 were analyzed. The capsule serotyping, multilocus sequence typing, and antimicrobial resistance analyses were performed on all the invasive GBS isolates, and whole-genome sequencing (WGS) was performed specifically on the type Ib GBS strain. A total of 188 neonates with invasive GBS disease during the study period were identified. The type Ib GBS strain accounted for 7.4% (*n* = 14) of neonatal GBS invasive diseases. Almost all type Ib GBS isolates belonged to sequence type 12 (13/14, 92.9%) and clonal complex 12. Neonates with type Ib GBS disease had a significantly higher rate of complicated sepsis (10/14, 71.4%; *P* < 0.05) and sepsis-attributable mortality (6/14, 42.9%; *P* < 0.05). Additionally, type Ib GBS isolates had significantly higher rates of resistance to erythromycin and clindamycin (both 100%; *P* < 0.05) than other GBS serotypes. WGS revealed the presence of an ~75-kb integrative and conjugative element, ICE*Sag37*, comprising multiple antibiotic resistance and virulence genes, and PI-1 plus PI-2a were noted in all type Ib serotype 12 (ST12) GBS isolates; these isolates may be responsible for its high invasiveness and antimicrobial resistance rates. The genomic characteristics of the type Ib clonal complex 12 (CC12) GBS strain may account for the high illness severity associated with this strain and its antibiotic resistance. Continuous monitoring and advanced strategies to control the spread of type Ib CC12 GBS should be considered.

**IMPORTANCE** A type Ib ST12 GBS strain is not a common isolate in neonatal invasive diseases and has been ignored for a long time. However, the recent literature and our data showed that such a GBS strain is associated with a significantly higher risk of severe sepsis, higher illness severity, and a significantly higher rate of sepsis-attributable mortality. This study found a novel gene cluster, including the presence of ICE*Sag37* and specific pilus genes, carrying multiple antimicrobial resistance and virulence genes, which may be responsible for the clinical characteristics. Because of the higher mortality and severity of illness, we concluded that continuous monitoring of the type Ib ST12 GBS strain is warranted in the future.

## INTRODUCTION

Streptococcus agalactiae (also known as group B Streptococcus [GBS]) is an important cause of neonatal late-onset sepsis and meningitis (1–3). Invasive diseases caused by GBS in neonates are associated with a high mortality rate and lead to long-term neurological sequelae, especially among survivors of GBS meningitis (4, 5). Despite the wide application of maternal screening during pregnancy and intrapartum antibiotic prophylaxis, which have reduced the incidence of GBS early-onset disease (EOD, birth to the 7th day of life), the rate of GBS late-onset disease (LOD; disease occurring after 8 days of age) remains unchanged (6–8). Recent studies have focused on the development of vaccines based on the molecular characteristics of GBS isolates and virulence and genetic mechanisms that lead to severe GBS-associated sepsis and meningitis (9–13).

GBS isolates are traditionally categorized based on 10 different capsular serotypes and numerous sequence types (STs) after multilocus sequence typing (MLST) of seven housekeeping genes in epidemiological studies (6, 10, 14). Genetically related STs can be clustered into clonal complexes (CCs) after phylogenetic analyses (13, 14). These subgroupings aid in the development of vaccines, the investigation of genetic evolution, and the tracking of transmission routes and probable clonal expansion and potentially contribute to preventive strategies (15–17). Although the type III CC17 GBS strain is well known to have caused the majority of cases of neonatal sepsis and meningitis in recent decades (17–19), the serotype Ib GBS strain was recently reported to be emerging with high incidence in Asia, especially in southeast China and Japan (20–23). The type Ib GBS strain is worthy of continuous monitoring due to its multidrug resistance and considerably high association with severe sepsis (20–23), but the relevant data regarding its molecular and genetic properties remain limited. We aimed to investigate the clinical and molecular characteristics of type Ib GBS isolates that cause severe invasive diseases in neonates.

## RESULTS

### Clinical characteristics of neonates with invasive GBS disease.

During the study period, there were a total of 188 neonates with invasive GBS disease. The median (interquartile range [IQR]) gestational age and birth body weight (BBW) of the cohort were 38.0 (36.3 to 39.0) weeks and 2,885 (2,550 to 3,235) g, respectively. Most cases (141/188, 75.0%) were term-born (gestational age [GA], ≥37 weeks) neonates, only 5.3% (*n* = 10) were extremely preterm (GA, ≤28 weeks), and 8.0% (*n* = 15) were very-low-birthweight infants (BBW, <1,500 g). There were 44 cases of EOD, 133 cases of LOD, and 11 cases of late LOD (LLOD). In this cohort, the type III ST17 GBS strain accounted for the highest proportion of invasive diseases (67.0%, *n* = 126), followed by types Ia (16.5%, *n* = 31), Ib (7.4%, *n* = 14), and V (3.7%, *n* = 7). The patients’ demographics, clinical manifestations, and laboratory findings are summarized in Table 1.

**TABLE 1:** Patient demographics and clinical manifestations of neonatal invasive GBS infections in Chang Gung Memorial Hospital, 2003 to 2020

Demographic or clinical manifestation	Type Ib GBS sepsis (total *n* = 14)	Type III GBS sepsis (total *n* = 126)	Other serotypes (total *n* = 48)	*P* values^*a*^
Gestational age (wks)	37.5 (35.5–39.0)	38.5 (36.5–39.0)	37.0 (35.0–39.0)	0.106, 0.317
Birth body wt (g)	2,785.0 (2,490–31,20)	2,960.0 (2,580–3,350)	2,685.0 (2,350–3,050)	0.112, 0.643
Gender (male/female) [*n* (%)]	6 (42.9)/8 (57.1)	49 (38.9)/77 (61.1)	26 (42.0)/22 (58.0)	0.780, 0.550
Birth by NSD or Cesarean section [*n* (%)]	9 (64.3)/5 (35.7)	93 (73.8)/33 (26.2)	28 (71.4)/20 (28.6)	0.528, 0.765
5-min Apgar score of <7 [*n* (%)]	2 (14.3)	3 (2.4)	7 (14.9)	0.023, 0.978
Premature rupture of membrane [*n* (%)]	7 (50.0)	11 (8.7)	17 (35.4)	<0.001, 0.363
Onset of GBS bacteremia (day), [median (IQR)]	4.5 (1.0–50.3)	30.0 (15.8–56.0)	15.5 (1.0–40.0)	0.038, 0.759
Early-onset sepsis (≤7 days) [*n* (%)]	8 (57.1)	14 (37.7)	22 (45.8)	<0.001, 0.352
Late-onset sepsis (8–90 days) [*n* (%)]	4 (28.6)	106 (58.0)	23 (47.9)	<0.001, 0.357
Very-late onset sepsis (>90 days) [*n* (%)]	2 (14.3)	6 (4.3)	3 (6.3)	
Clinical features^*b*^ [*n* (%)]				
Fever (≥38.3°C)	5 (35.7)	116 (92.1)	32 (66.7)	<0.001, 0.038
Apnea, bradycardia and/or cyanosis	12 (85.7)	32 (25.4)	23 (47.9)	<0.001, 0.015
Ventilator requirement				<0.001, <0.001
Room air or nasal canuala	2 (14.3)	98 (77.8)	24 (50.0)	
Noninvasive ventilator (N-CPAP and N-IMV)^*c*^	1 (7.1)	10 (7.9)	8 (16.7)	
Intubation with conventional ventilator	7 (50.0)	16 (12.7)	13 (27.1)	<0.001, <0.001
High-frequency oscillatory ventilator	4 (28.6)	2 (1.6)	3 (6.3)	<0.001, <0.001
Abdominal distension and/or vomiting	10 (71.4)	38 (30.2)	23 (47.9)	0.097, 0.141
Hypoglycemia	5 (35.7)	9 (7.1)	8 (16.7)	0.006, 0.146
Hypotension	8 (57.1)	11 (8.7)	15 (31.3)	<0.001, 0.116
Severe sepsis	10 (71.4)	39 (31.0)	17 (35.4)	<0.001, 0.030
Disseminated intravascular coagulopathy	4 (28.6)	6 (4.8)	3 (6.3)	<0.001, 0.020
Requirement of blood transfusion^*d*^	9 (64.3)	53 (42.1)	26 (54.2)	0.156, 0.555
Laboratory data at onset of GBS bacteremia [*n* (%)]				
Leukocytosis (WBC > 20,000/liter)	8 (57.1)	78 (61.9)	24 (50.0)	0.777, 0.764
Leukopenia (WBC < 4,000/liter)	2 (14.3)	28 (22.2)	11 (22.9)	0.492, 0.485
Shift to left in WBC (immature > 20%)	2 (14.3)	15 (11.9)	6 (12.5)	0.796, 0.861
Anemia (hemoglobin level < 11.5 g/dL)	4 (28.6)	66 (52.4)	27 (56.3)	0.091, 0.068
Thrombocytopenia (platelet < 150,000/μL)	9 (64.3)	12 (9.5)	10 (20.8)	<0.001, 0.004
Metabolic acidosis	8 (57.1)	8 (6.3)	11 (22.9)	<0.001, 0.005
Coagulopathy	8 (57.1)	9 (7.1)	13 (27.1)	<0.001, 0.054
C-reactive protein (mg/dL) [median (IQR)]	65.0 (19.0–135.8)	29.5 (8.0–105.3)	41.5 (15.0–78.0)	<0.097, 0.300
Final in-hospital mortality [*n* (%)]	6 (42.9)	5 (4.0)	8 (16.7)	<0.001, 0.049

a *P* values are for comparisons between type Ib GBS strains versus type III GBS strains and type Ib GBS strains versus other serotypes.

b At onset of GBS bacteremia.

c N-CPAP, nasal continuous positive airway pressure; N-IMV, non-invasive mechanical ventilation.

d Indicating leukocyte-poor red blood cell and/or platelet transfusion.

We found that neonates with type Ib GBS sepsis had no significant differences regarding the patients’ demographics and underlying chronic comorbidities compared with those infected by other GBS serotypes. Patients with type Ib GBS isolates were more likely to be EOD (57.1%) and presented with more severe clinical manifestations, including a higher likelihood of respiratory failure requiring mechanical ventilation, septic shock, and severe sepsis (Table 1). Neonates with type Ib GBS disease had a significantly higher rate of complicated sepsis (10/14, 71.4%; *P* < 0.05) and sepsis-attributable mortality (6/14, 42.9%; *P* < 0.05) than those with other serotypes, although the type III GBS strain was the most common cause of neonatal meningitis (*n* = 29, 68.8%). The overall mortality rate of the cohort was 10.1% (a total *n* of 19 died), and 31.6% (*n* = 6) of the mortality cases were type Ib GBS sepsis.

### Antimicrobial resistance profile of GBS isolates.

All GBS isolates were susceptible to penicillin, vancomycin, teicoplanin, and cefotaxime. Overall, the antibiotic resistance rates to erythromycin and clindamycin were 49.5% and 48.9%, respectively. However, type Ib and III GBS isolates had significantly higher rates of resistance to erythromycin and clindamycin (90.1% and 72.6%, respectively; all *P* < 0.05) than other serotypes. It is worth noting that 100% of all type Ib GBS isolates in our cohort were resistant to both erythromycin and clindamycin.

### Molecular characteristics of the type Ib CC12 GBS isolate.

The MLST analyses showed that most of the type Ib GBS isolates obtained from our cohort were ST12 (13/14, 92.9%), and only one was ST10. The relationships between clonal complexes, sequence types, and serotypes are summarized in Table 2. Type III CC17 GBS accounted for nearly three-fourths of our cohort, and an increasing trend of the type III CC17 GBS strain has been reported since 2012.

**TABLE 2:** Relationships between sequence types, clonal complexes, and serotypes of all S. agalactiae isolates causing neonatal invasive diseases in CGMH, 2003 to 2020

Serotype (total *n* = 188)	*N* (%)	CC	ST	*N* (%)	Meningitis [*n* (%)]	Mortality [*n* (%)]
Ia	31 (16.5)	CC1	ST1	1 (3.2)	0	0
		CC12	ST268	2 (6.5)	2 (100)	0
		CC23	ST23	15 (48.4)	5 (33.3)	2 (13.3)
		CC24	ST24	9 (29.0)	3 (33.3)	1 (11.1)
		CC144	ST144	1 (3.2)	0	0
		CC890	ST890	3 (9.7)	0	0
Ib	14 (7.4)	CC12	ST10	1 (7.1)	0	1 (100)
			ST12	13 (92.9)	4 (30.8)	5 (38.5)
II	4 (2.1)	CC1	ST1	4 (100)	1 (25.0)	2 (50.0)
III	126 (67.0)	CC17	ST17	113 (89.7)	27 (23.9)	5 (4.4)
		CC19	ST19	10 (7.9)	5 (50.0)	0
			ST335	1 (0.8)	0	0
		CC438	ST438	1 (0.8)	1 (100)	0
		CC890	ST890	1 (0.8)	0	0
IV	1 (0.5)	CC23	ST452	1 (100)	0	0
V	7 (3.7)	CC1	ST1	4 (57.1)	0	1 (25.0)
		CC12	ST12	1 (14.3)	0	0
		CC23	ST23	1 (14.3)	0	1 (100)
		CC890	ST890	1 (14.3)	0	0
VI	5 (2.7)	CC1	ST1	5 (100)	0	1 (20.0)

Whole-genome sequencing (WGS) was performed using a total of one Ib ST12 strain (N92), one Ib ST10 strain, and one ST17 III (N5) GBS isolate, based on their sources and the patient demographics. Strains NGBS128 and CP019978, both Ib ST12 GBS isolates from neonates with invasive GBS diseases, were used as the reference strains (24). Another Ib ST10 GBS reference strain (NZ_SBIB000000000.1) from NCBI was also used as the reference strain for comparison. Comparative genome analyses were performed for all the GBS isolates and the two reference strains to track the possible genomics of the mobile elements and insertion sequences (ISs). All the genes related to the component systems CovS/R, antibiotic resistance, pilus formation, capsular serotype, and virulence were investigated.

**FIG 1:**
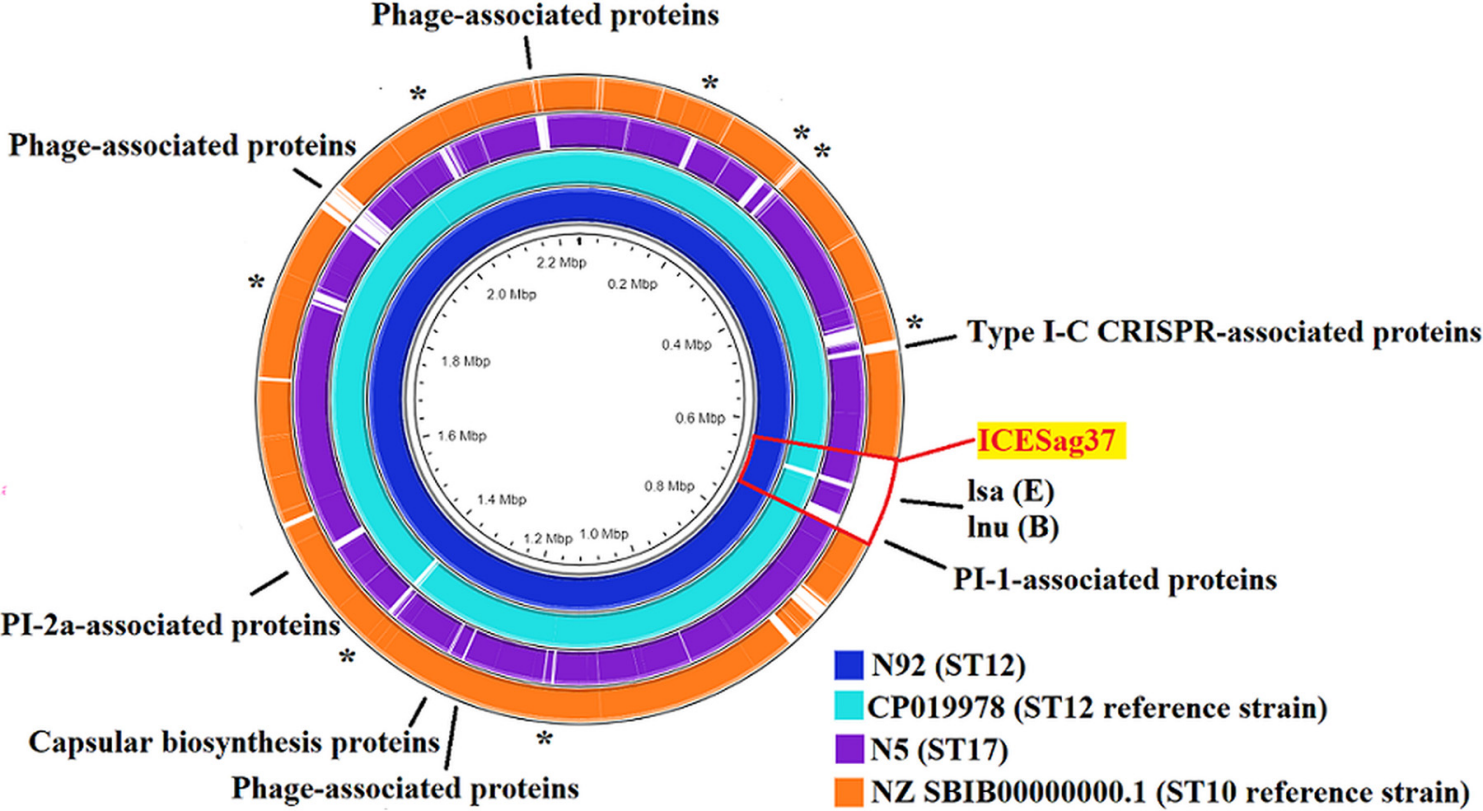
Whole-genome analysis of one type Ib ST12 GBS strain (N92), one type III ST17 GBS strain (N5), and two reference strains, CP019978 (type Ib ST12) and NZ SBIB00000000.1 (type Ib ST10). The genome scales, in megabase pairs of these two reference strains, are given in the innermost circle. TBLASTN comparisons of the genomes of the reference GBS strains with the complete genomes of the type Ib ST12 and antibiotic-susceptible type III ST17 GBS strains are shown in different colors. The genomes of the two-component system CovS/R and genes related to capsular serotypes, pili, virulence, and antibiotic resistance are presented in different colors. Several genes encoding several phage-associated proteins, PI-1- and PI-2a-associated proteins and type I CRISPR-associated proteins, are present in type Ib ST12 GBS strains only. *, genes encoding hypothetical protein.

**FIG 2:**
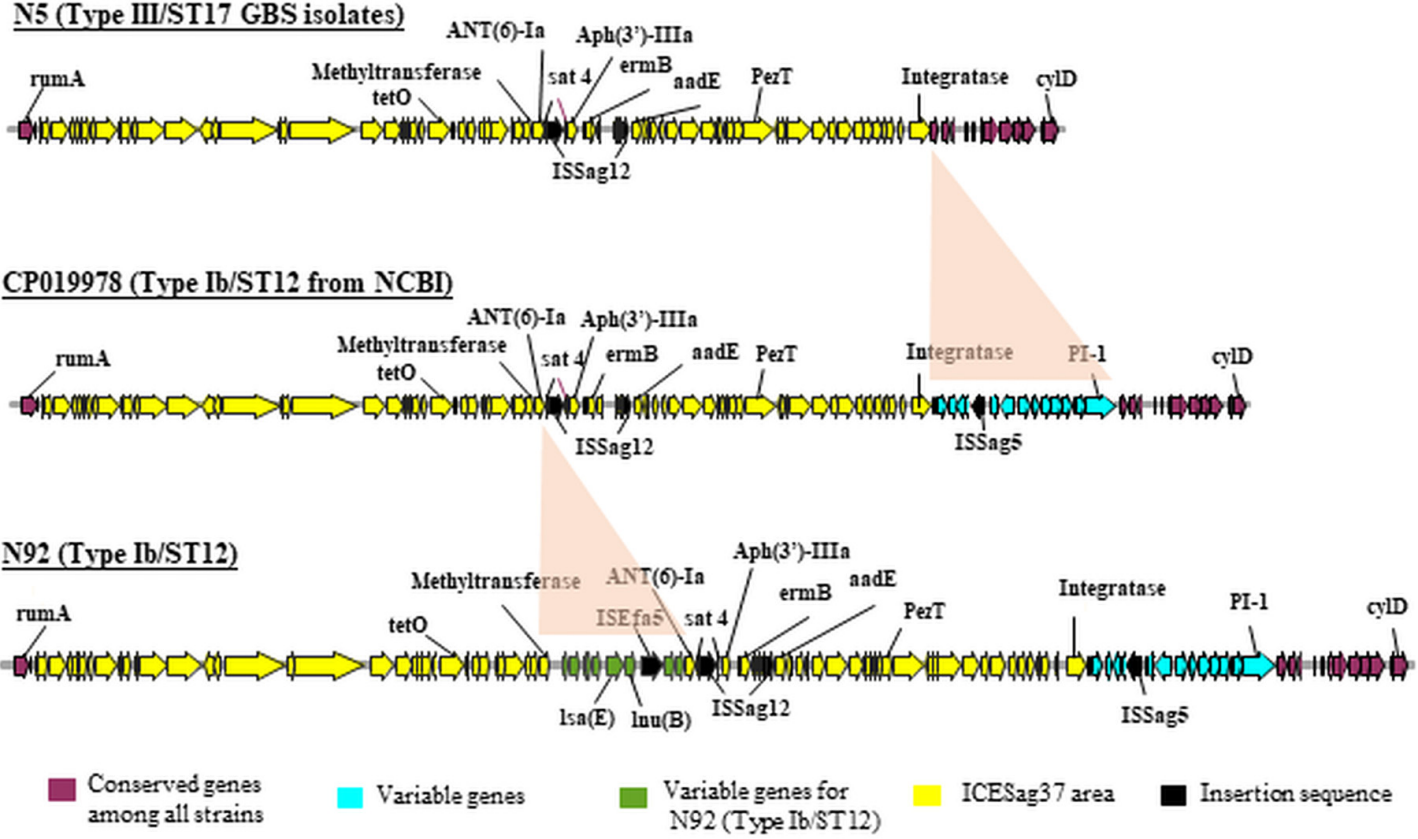
The site-specific integration of ICE*Sag37*, starting from the beginning of the *rum*A sequence to the end of the integrase sequence, is clearly visible in the type Ib ST12 (both clinical isolate and reference strain) and type III ST17 GBS strains, but not in the type Ib ST10 GBS strain. The integrative and conjugative element ICE*Sag37*, which carries multiple antimicrobial genes and virulence genes, is shown in yellow. Comparison of the clinical type Ib ST12 GBS isolate (N92) and the reference strain CP019978 shows additional *lsa*(E) and *lun*(B) genes (green color) inside the integrative and conjugative element ICE*Sag37*. Additionally, the loss of PI-1 and IS*Sag5* is replaced by the acquisition of ICE*Sag37* in antimicrobial-resistant ST-17 type III GBS strains. Conserved genes are indicated in purple, and variable genes are indicated in light blue and green (for N92). Antimicrobial resistance genes are indicated.

The genes of component systems CovS/R, most antimicrobial resistance genes, and most virulence genes were not significantly different between type Ib ST12, type Ib ST10, and type III ST17 GBS strains (Fig. 1). The genes encoding several phage-associated proteins, PI-1- and PI-2a-associated proteins, and type I CRISPR-associated proteins were significantly different between the Ib ST12, Ib ST10, and III ST17 GBS strains. After searching and performing comparisons using the ICEfinder database (https://db-mml.sjtu.edu.cn/ICEfinder/ICEfinder.html), both type Ib ST12 and type III ST17 strains exhibited the integrative and conjugative element ICE*Sag37*, an element that carries multiple antibiotic resistance genes and virulence genes, but it was not present in the type Ib ST10 GBS strain. Additionally, the insertion sequence IS*Sag*5 was found upstream of ICE*Sag37* in all Ib ST12 GBS strains but was not found in type III CC17 GBS strains (Fig. 2).

We previously found the components and characterization of ICE*Sag37* to be similar to that found by Zhou et al. in a type Ib ST12 GBS strain isolated from a septic neonate (25). The size of ICE*Sag37* was 73,429 kb, and there were numerous genes for virulence, integrase, MobC and relaxases, a two-component signal transduction system (*nisK*/*nisR*), and multiple antibiotic resistance genes, including *erm*(B) (resistance gene to erythromycin), *tet*(O) (resistance gene to tetracycline), and *aadE*, *aphA*, and *ant-6* (resistance genes to aminoglycosides) within ICE*Sag37*. An important virulence gene, *pezT*, was integrated in the mosaic area of the antibiotic resistance genes of ICE*Sag37* (Fig. 2). Interestingly, there were the additional antibiotic resistance genes *isa*(E) and *lun*(B) within ICE*Sag37* in our clinical type Ib ST12 GBS isolates, unlike in the reference strain CP019978, as shown in Fig. 2.

**FIG 3:**
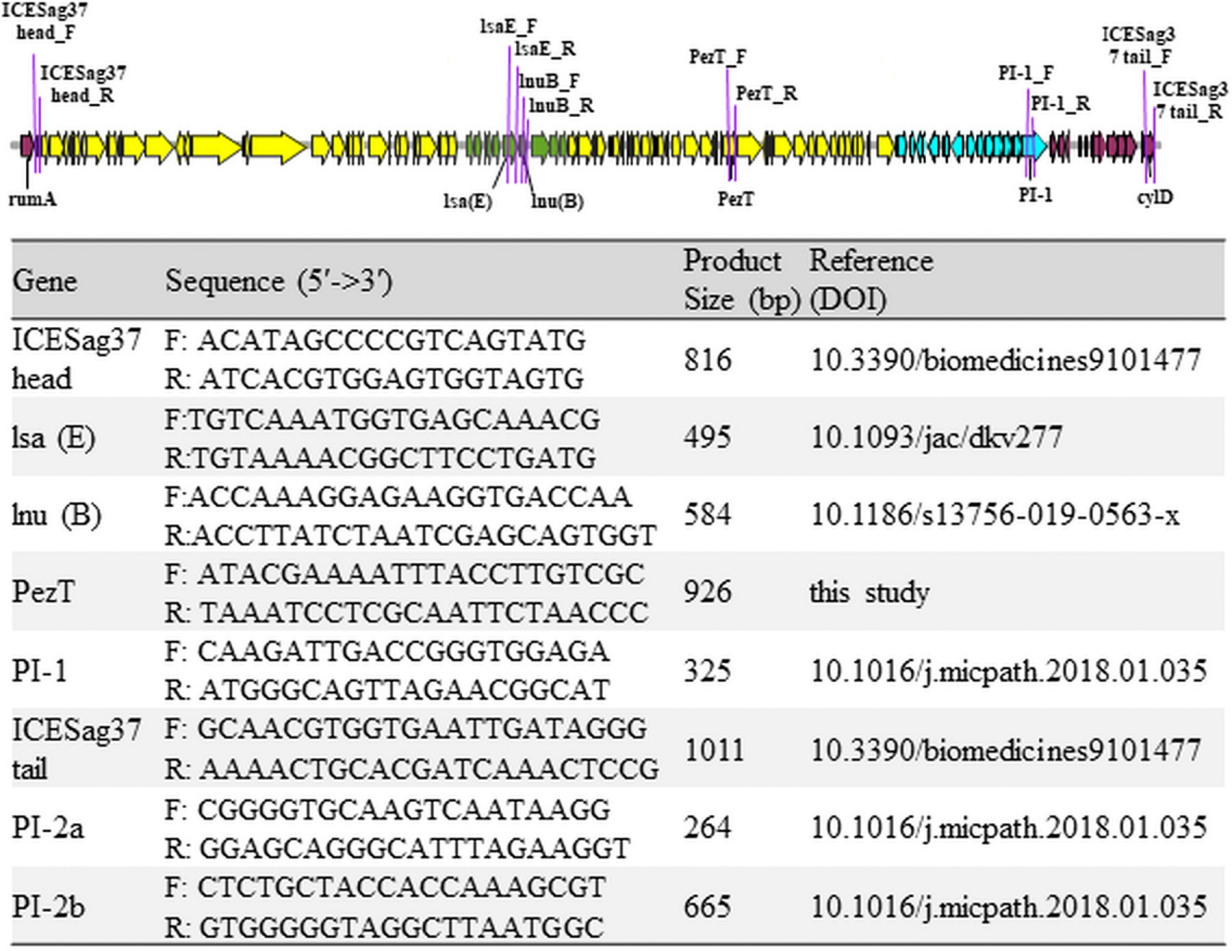
Primers used for targeted genes in PCR and the relative positions of these genes inside or around the integrative and conjugative element, ICE*Sag37*, in type Ib ST12 GBS isolates.

PCR was performed for all type Ib and type III GBS isolates to verify the results and confirm the presence of multiple genes in all clinical GBS isolates. The primers of all targeted genes and the relative positions inside or around ICS*Sag37* are summarized in Fig. 3. We found that 92.3% (12 of 13) of Ib ST12 GBS isolates had ICE*Sag37*, PI-1 plus PI-2a, and the virulence gene *pezT*, which showed a high degree of genetic homogeneity among the type Ib CC12 GBS isolates (Table 3).

**TABLE 3:** Antibiotic susceptibility patterns, pilus distribution, and presence of IS*Sag5*, ICE*Sag37*, and numerous antibiotic resistance genes in all type Ib ST12 and type III ST17 GBS isolates

Category	No. (%) of GBS isolates that were:					
	Type Ib ST12 (total *n* = 13)			Type III ST17 (total *n* = 113)		
Pilus genes	PI-1 + PI-2a	PI-1 + PI-2b	PI-2a only	PI-1 + PI-2a	PI-1 + PI-2b	PI-2b
Total *n* (%)	12 (92.3)	0	1 (7.7)	0	15 (13.3)	98 (86.7)
Virulence genes						
*pezT*	12 (100)	0	1 (100)	0	0 (0)	98 (100)
*hvgA*	0 (0)	0	0 (0)	0	15 (100)	98 (100)
Antibiotic resistance genes						
*lsa*(E)	6 (50.0)	0	0 (0)	0	0 (0)	26 (26.5)
*lun*(B)	6 (50.0)	0	0 (0)	0	0 (0)	26 (26.5)
ICE*Sag37*	12 (100)	0	1 (100)	0	0 (0)	98 (100)
IS*Sag5*	12 (100)	0	1 (100)	0	15 (100)	0 (0)
Antibiotic resistance pattern^*a*^						
Ery (R) + Clin (R)	12 (100)	0	1 (100)	0	3 (20.0)	95 (96.9)
Ery (S) + Clin (S)	0	0	0	0	7 (46.7)	3 (3.1)
Ery (R) + Clin (S)	0	0	0	0	5 (33.3)	0
Ery (S) + Clin (R)	0	0	0	0	0	0

a Data are the number (percent) of resistant GBS isolates. Ery, erythromycin; Clin, clindamycin; R, resistant; S, susceptible. All GBS isolates were susceptible to vancomycin, teicoplanin, ampicillin, penicillin, and cefotaxime.

## DISCUSSION

The serotype Ib GBS strain is especially prevalent in Asian countries, accounting for 19.8 to 28.7% of all invasive GBS diseases in neonates, nonpregnant women, or a mixed population of pregnant women and neonates in recent reports (7, 15, 16, 20–23). Our data showed that the type Ib CC12 GBS strain was associated with a significantly higher rate of severe sepsis and sepsis-attributable mortality than other serotypes. Similar to the hypervirulent, antimicrobial-resistant type III CC17 GBS strain, the acquisition of ICE*Sag37*, which comprises multiple genes conferring antibiotic resistance and virulence, in a specific locus was noted in all of our type Ib ST12 GBS isolates. Although these isolates were obtained from clinical collection for nearly 2 decades, all type Ib ST12 GBS isolates in our cohort had ICE*Sag37*, IS*Sag5*, and *pezT*. Therefore, our comparative genome analysis showed that the type Ib ST12 GBS isolates exhibited a high degree of genetic homogeneity and may have been from the same clonal expansion.

Although the type III CC17 GBS strain is known to have caused the majority of neonatal invasive diseases in recent decades (8–10, 12, 13), the type Ib CC12 GBS strain has been found to be more virulent and fatal than the type III CC17 strain (20, 22, 24, 26). Recent reports found a mortality rate of 30.8% in neonates with type Ib CC12 GBS invasive disease (24), which was much higher than the reported 8.8 to 12% mortality rate associated with GBS invasive diseases caused by other serotypes, including a mortality rate of 12.5 to 18.5% associated with the type III CC17 GBS strain (8–10, 12, 13, 27–29). Zhang et al. and others also found that type Ib CC10 GBS isolates were associated with a significantly higher rate of meningitis and higher illness severity (20–22). Of the six mortality cases in this cohort, a fulminant course was noted despite appropriate antibiotics being administered on time, and most of these patients were term-born neonates without chronic comorbidities. This finding may be explained by the findings observed in a recent *in vivo* model that showed that the type Ib CC12 GBS strain exhibits strong invasiveness and resistance to phagocytes (24). Liu et al. (24) found that in the circulatory system, it was more difficult to eradicate the type Ib CC12 GBS strain than the type III ST17 strain. Therefore, new and aggressive therapeutic strategies, including close monitoring of clinical deterioration and development of novel anti-inflammatory agents, are warranted for neonates with type Ib CC12 GBS diseases.

Both ST10 and ST12, founders of CC10 and CC12, are related to serotype Ib GBS strains (20, 22, 24). While some studies have shown that most type Ib GBS isolates were ST10 (20, 22), other reports have shown that ST12 isolates outnumbered ST10 isolates (7, 24, 25). Most of the type Ib GBS isolates obtained from our cohort were ST12 instead of ST10, and significant differences in genetic characteristics between the ST12 GBS strain and the ST10 GBS isolates were observed. The major differences were the presence of ICE*Sag37*, some phage-associated proteins, and type I-C CRISPR-associated proteins in ST12 GBS isolates that were absent in ST10 GBS isolates. Most of the phage-associated and type I-C CRISPR-associated proteins contributed to the adaptive and invasive capabilities of the GBS isolates (see Table S1 in the supplemental material). Additionally, because ICE*Sag37* carries multiple virulence genes and antibiotic resistance genes, the type Ib ST12 GBS strain is supposed to be more virulent and fatal than the type Ib ST10 GBS strain, which has been reflected by the significantly higher mortality rate associated with type Ib ST12 GBS sepsis than with type Ib ST10 GBS sepsis in neonates (20, 22, 24).

A various group of mobile gene elements are integrated into the ICE, which then integrates into the host chromosome. ICEs play an important role in carrying several phenotypical genes and contribute to bacterial evolution and adaptation after genetic integration. Zhou et al. (30) was the first to use a type Ib ST12 GBS strain (the S. agalactiae Sag37) and documented the presence of ICE*Sag37* with numerous antibiotic resistance genes in the isolate. Zhou et al. found that the sequences of ICE*Sag37* were highly homologous and similar to an ~54-kb ICE from the S. agalactiae 2603 V/R strain and then categorized it as an ICE*Sa2603* family-like ICE (30, 31). In addition, the antibiotic resistance gene cluster of ICE*Sag37* was found to be similar to a plasmid in Enterococcus faecalis, which indicates the possibility of interspecies genetic exchange (30). Although several ICEs are not uncommon in various GBS strains, ICE*Sag37* is only found in the hypervirulent type Ib ST12 and type III ST17 GBS isolates. Because of the presence of multiple antibiotic resistance genes in ICE*Sag37*, it is hypothesized that the characteristic of high antibiotic resistance is associated with its high virulence, invasiveness, and more severe clinical manifestations. Additionally, the high degree of genetic homogeneity among our type Ib CC12 GBS isolates, which were collected over almost 2 decades, showed less genetic recombination and possible clonal expansion.

Pili are known to play an important role in the progression of sepsis and meningitis through their role in cell adhesion, transcytosis, and enhanced penetration of the blood-brain barrier (32, 33). At least one of the three pilus variants (types 1, 2a, and 2b) can be found in all GBS strains, and specific GBS strains or phylogenetic lineages have specific GBS pilus type profiles (33–35). For example, a combination of PI-1 and PI-2b genes is mostly found in type III CC17 GBS isolates (34, 35), and our data showed that most of the type Ib GBS isolates had PI-I plus PI-2a, which was similar to previous reports (20, 22, 24). We previously found that loss of PI-1 and presence of ICE*Sag37* correlated with clonal expansion of the antibiotic resistance type III CC17 GBS strains to predominate in our cohort (36). However, the type Ib CC12 GBS isolates obtained in our cohort had both PI-1 and ICE*Sag37*, and the additional effects of PI-1 plus ICE*Sag37* on virulence remain unknown and deserve further study.

The *pezT* gene was initially found in Streptococcus pneumoniae and is part of the epsilon/zeta system (the PezA/T system), which is a special toxin/antitoxin system to protect the bacterium itself (37, 38). PezT can activate the release of specific toxins to attack the host cells or competing microorganisms, which has been considered its pathogenesis and invasive mechanisms (37, 38). Our data are consistent with a previous study that found the PezA/T system is unique in the CC12 GBS strain and it plays an important role in virulence and bacterial pathogenicity (24). Therefore, the PezA/T system is presumed to be associated with the higher illness severity of the CC12 GBS strain, and it deserves further investigation.

There were some limitations in this study. All invasive GBS isolates were from a single center in Taiwan, and the number of cases of type Ib CC12 GBS that caused neonatal invasive diseases was small. Further large-scale, multicenter studies are warranted to investigate the clinical and genetic diversities of the type Ib GBS strain. The expression of genes was not investigated in this study, and only the PCR method was used to investigate the molecular characteristics of our GBS isolates. Additionally, we lost some early mortality cases and some invasive GBS strains more than 10 years ago, and these cases were not included in this study.

In conclusion, the type Ib CC12 GBS strain is characterized by more severe clinical manifestations, a high antimicrobial resistance rate, and a significantly higher rate of mortality than other serotypes. Genetically, type Ib CC12 GBS isolates have ICE*Sag37*, which carries numerous antibiotic resistance genes and specific virulence profiles and may be responsible for the higher illness severity and worse outcomes associated with infections with this strain. Given the clinical importance and genetic specificity, further large-scale studies in which more cases are enrolled and type Ib GBS isolates are obtained and continuously monitored for potential genetic recombination are warranted in the future.

## MATERIALS AND METHODS

### GBS isolates, data collection, and definition.

Between January 2003 and December 2020, all patients with invasive GBS diseases who were hospitalized in Linkou Chang Gung Memorial Hospital (CGMH), a tertiary-level medical center in northern Taiwan, were enrolled for analyses. Invasive GBS disease was defined as GBS infection with GBS strains isolated from a sterile site, including blood, cerebrospinal fluid (CSF), and pleural or peritoneal fluids. Patients with GBS isolates isolated from sputum or bronchoalveolar lavage fluid were not enrolled. Data regarding all GBS isolates were retrieved from the bacterial library of CGMH’s central laboratory. The electronic records containing patients’ demographic information, clinical characteristics, hospital courses, treatment, and outcomes were also reviewed and recorded on a standard form.

This study was approved by the Institutional Review Board of CGMH (IRB 202002475B0), and a waiver for informed consent for anonymous data collection was approved.

Meningitis was defined based on the World Health Organization as the presence of clinical signs of possible serious bacterial infection (2, 39) and CSF culture positive for bacterial pathogens or blood culture, PCR, or latex agglutination tests positive for bacterial pathogens with a CSF leukocyte count of >20 × 10^6^/liter and a clinical presentation compatible with bacterial meningitis. For patients with severe sepsis and uncomplicated bacteremia, we applied the definitions provided by the Centers for Disease Control and Prevention (40). The presence of neurological complications and long-term neurological sequelae in these patients was evaluated based on the definitions used in previous studies (4, 41). Early-onset disease (EOD), late-onset disease (LOD), and late LOD (LLOD) were defined as disease onset between 0 and 7 days, between 8 and 90 days, and after 90 days of life, respectively (20–22).

### Capsular serotyping and MLST.

The multiplex PCR assay was used to analyze the capsular serotypes to identify GBS isolates of types Ia to IX. The DNA isolation method and the PCR assay through which seven housekeeping genes (*adhP*, *atr*, *glcK*, *glnA*, *pheS*, *sdhA*, and *tkt*) were amplified and sequenced were based on a standard protocol and have been described in our previous publications (10). MLST was then conducted based on the standard procedure described in our previous studies (42). After PCR, the sequence type (ST) was assigned based on the allelic profile of each fragment and determined via the Streptococcus agalactiae MLST database (http://pubmist.org/sagalactiae). All GBS isolates could be clustered into several major clonal complexes (CCs) based on the goeBURST program (43).

### Antimicrobial susceptibility testing.

Antimicrobial susceptibility testing was performed with the disk-diffusion method as described for previous studies (43). The double-disk-diffusion test was applied to identify inducible clindamycin resistance. All GBS isolates were rated for susceptibility to seven antibiotics, including erythromycin, penicillin, clindamycin, vancomycin, ampicillin, cefotaxime, and teicoplanin, according to the guidelines of the Clinical and Laboratory Standards Institute (CLSI) for the disk-diffusion method (44).

### Whole-genome sequencing.

Three isolates were selected from the type Ib ST12 (termed N92) and type III ST-17 (termed N48, N96, and N5) GBS strains. We used the lysozyme-sodium dodecyl sulfate-proteinase K method to extract the DNA. WGS was performed using both PacBio SMRT (Pacific Biosciences, Menlo Park, CA, USA) (26) and MiSeq (Illumina, San Diego, CA, USA) (45) sequencing technologies. The sequencing library was prepared using a TruSeq DNA LT sample prep kit (Illumina, San Diego, CA, USA) for the Illumina MiSeq system. Genomic libraries were generated using Nextera XT kits (Illumina, San Diego, CA, USA). We used SPAdes (version 3.9.0) to assemble the sequence. All genome sequences were subjected to BLAST analysis using the NCBI genome database to identify possible plasmid sequences. After the *de novo*-assembled genome was generated, Prokka (version 1,12) (46) was used for genome annotation and the identification of rRNA-encoding and tRNA-encoding regions.

### Statistical analysis.

Because type III GBS neonatal sepsis is well known as an emerging disease, we compared neonates with type Ib GBS sepsis with those with type III GBS sepsis and infections with other serotypes. Categorical and continuous variables were expressed as proportions and the median (in parentheses, the IQR), respectively. Categorical variables were compared using the χ^2^ test or Fisher’s exact test; odds ratios and 95% confidence intervals were calculated. Continuous variables were compared using the Mann-Whitney *U* test and the *t* test, depending on the distributions. The trend of the proportions of the categorical variables among the subgroups was analyzed by the Cochran-Armitage trend test. Results with *P* values of <0.05 were considered statistically significant. All statistical analyses were performed using SPSS version 23 (IBM SPSS Statistics).

### Ethics statement.

This study was approved by the Institutional Review Board of Chang Gung Memorial Hospital, with a waiver of informed consent because all patient records and information were anonymized and deidentified prior to analysis.

### Data availability.

The data sets used or analyzed during the current study are available from the corresponding author on reasonable request.
