# Supplementary material for: Clonal Complex 12 Serotype Ib Streptococcus agalactiae Strain Causing Complicated Sepsis in Neonates: Clinical Features and Genetic Characteristics
Source: Microbiol Spectr. 2022 Dec 8;11(1):e03778-22. doi: 10.1128/spectrum.03778-22 (PMC9927456; doi:10.1128/spectrum.03778-22)
Supplement: Supplemental file 1 — Supplemental material. Download spectrum.03778-22-s0001.pdf, PDF file, 0.01 MB [file spectrum.03778-22-s0001.pdf]

Supplemental Table 1. The phage-associated proteins of type Ib/ST12 GBS isolates and the functions

**Phage-associated proteins**	**Function**
Hic A/B family protein	phage resistance
phage holin	control the length of the infective cycle for lytic phages
phage tail protein	construction of phage
phage tail tape measure protein	implicated in genome injection
phage capsid protein	construction of phage
peptidase U35	construction of phage
phage portal protein	form a channel for bidirectional passage of viral DNA
HNH endonuclease	key components of phage DNA packaging machines
DUF3310 domain-containing protein	unknown function
ImmA/IrrE family metallo-endopeptidase	regulation of horizontal gene transfer
ArpU family transcriptional regulator	autolysin regulatory
phage anti-repressor protein	inhibit the action of other prophage repressor proteins
phage repressor protein	control the genetic switch that determines whether a lytic or lysogenic cycle will follow infection
30S ribosomal protein S4	Anti-termination factor
